# Effectiveness of perampanel as the only add‐on: Retrospective, multicenter, observational real‐life study on epilepsy patients

**DOI:** 10.1002/epi4.12649

**Published:** 2022-09-22

**Authors:** Sara Gasparini, Edoardo Ferlazzo, Sabrina Neri, Vittoria Cianci, Alfonso Iudice, Francesca Bisulli, Paolo Bonanni, Emanuele Caggia, Alfredo D'Aniello, Carlo Di Bonaventura, Jacopo C. DiFrancesco, Elisabetta Domina, Fedele Dono, Antonio Gambardella, Carla Marini, Alfonso Marrelli, Sara Matricardi, Alessandra Morano, Francesco Paladin, Rosaria Renna, Pasquale Striano, Angelo Pascarella, Michele Ascoli, Umberto Aguglia, PEROC Study Group, Arbasino Carla, Bagnasco Irene, Barbero Pierangelo, Bartolini Emanuele, Maria A. Bassetti, Belcastro Vincenzo, Beretta Simone, Boero Vanni, Bonuccelli Alice, Caggia Emanuele, Cantello Roberto, Casellato Susanna, Casula Natascia, Cesnik Edward, Ciampa Clotilde, Cipriani Anna Maria, Coletti Moja Mario, Conti Marta, Coppola Giangennaro, Corea Francesco, Crichiutti Giovanni, d’Orsi Giuseppe, Dainese Filippo, Danieli Alberto, De Curtis Marco, De Giorgis Valentina, Del Gaudio Luigi, Deleo Francesco, Di Gennaro Giancarlo, Giacomo Evangelista, Filipponi Stefania, Fortunato Francesco, Gagliano Attilio, Giacomini Thea, Gilio Francesca, Giordano Alfonso, Giuliano Loretta, Haggiag Shalom, Jensen Stella, Labate Angelo, Lupato Angelica, Macorig Greta, Mancardi Maria Margherita, Marino Daniela, Marrelli Alfonso, Mostacci Barbara, Muccioli Lorenzo, Negri Susanna, Operto Francesca, Orsini Alessandro, Palumbo Pasquale, Pascarella Mariagrazia, Peretti Alessia, Perri Gabriella, Pondrelli Federica, Pustorino Giuseppina, Ranzato Federica, Servo Serena, Siri Laura, Spitaleri Orazio, Stabile Andrea, Strigaro Gionata, Tinuper Paolo, Varesio Costanza

**Affiliations:** ^1^ Department of Medical and Surgical Sciences Magna Græcia University of Catanzaro Catanzaro Italy; ^2^ Regional Epilepsy Centre, Great Metropolitan “Bianchi‐Melacrino‐Morelli Hospital” Reggio Calabria Italy; ^3^ Institute of Molecular Bioimaging and Physiology, National Research Council Catanzaro Italy; ^4^ Department of Neurosciences, Section of Neurology University of Pisa Pisa Italy; ^5^ Department of Biomedical and NeuroMotor Sciences Alma Mater Studiorum‐University of Bologna Bologna Italy; ^6^ Epilepsy and Clinical Neurophysiology Unit Scientific Institute, IRCCS Eugenio Medea Treviso Italy; ^7^ Neurology Unit Oospedale Giovanni Paolo II Ragusa Italy; ^8^ IRCCS Neuromed Pozzilli Italy; ^9^ Epilepsy Unit, Department of Human Neurosciences "Sapienza" University of Rome Rome Italy; ^10^ Department of Neurology, ASST S. Gerardo Hospital University of Milano‐Bicocca Monza Italy; ^11^ U.C. Neurology, Ospedale Maggiore di Lodi ASST Lodi Italy; ^12^ Department of Neuroscience, Imaging and Clinical Science "G. D'Annunzio" University of Chieti‐Pescara Chieti Italy; ^13^ Neurologic Clinic Magna Græcia University of Catanzaro Catanzaro Italy; ^14^ Child Neurology and Psychiatric Unit, G. Salesi Pediatric Hospital United Hospitals of Ancona Ancona Italy; ^15^ Neurophysiopathology Unit, Epilepsy Center San Salvatore Hospital L'Aquila Italy; ^16^ Neurology Unit Epilepsy Center Venice Italy; ^17^ Unit of Neurology, Multiple Sclerosis Center Regina Elena National Cancer Institute, IFO Rome Italy; ^18^ IRCCS Istituto Giannina Gaslini Genova Italy; ^19^ Department of Neurosciences, Rehabilitation, Ophthalmology, Genetics, Maternal and Child Health University of Genova Genoa Italy; ^20^ Neurology Unit Marche Nord Hospital Pesaro Italy

**Keywords:** early add‐on, perampanel, real‐world, seizure freedom

## Abstract

**Objective:**

Perampanel (PER) is indicated as adjunctive antiseizure medication (ASM) in adolescents and adults with epilepsy. Data from clinical trials show good efficacy and tolerability, while limited information is available on the routine clinical use of PER, especially when used as only add‐on treatment.

**Methods:**

We performed an observational, retrospective, multicenter study on people with focal or generalized epilepsy aged >12 years, consecutively recruited from 52 Italian epilepsy centers. All patients received PER as the only add‐on treatment to a background ASM according to standard clinical practice. Retention rate, seizure frequency, and adverse events were recorded at 3, 6, and 12 months after PER introduction. Subanalyses by early or late use of PER and by concomitant ASM were also conducted.

**Results:**

Five hundred and three patients were included (age 36.5 ± 19.9 years). Eighty‐one percent had focal epilepsy. Overall, the retention rate was very high in the whole group (89% at 12 months) according with efficacy measures. No major differences were observed in the subanalyses, although patients who used PER as early add‐on, as compared with late add‐on, more often reached early seizure freedom at 3‐month follow‐up (66% vs 53%, *P* = .05). Treatment‐emergent adverse events occurred in 25%, far less commonly than in PER randomized trials.

**Significance:**

This study confirms the good efficacy and safety of PER for focal or generalized epilepsy in real‐life conditions. We provide robust data about its effectiveness as only add‐on treatment even in patients with a long‐standing history of epilepsy and previously treated with many ASMs.

**Key Points**

- Perampanel was effective as single add‐on to a previous antiseizure monotherapy.
- Retention rate was very high in the whole sample and in early add‐on population.
- Safety was good with few adverse events.

## 1 INTRODUCTION

Perampanel (PER) is a newly introduced antiseizure medication (ASM), highly selective, noncompetitive α‐amino‐3‐hydroxy‐5‐methyl‐4‐isoxazolepropionic acid (AMPA) receptor antagonist.^1^ To date, it is licensed for use as adjunctive therapy for focal onset seizures with or without evolution to bilateral tonic–clonic seizures and primary generalized tonic–clonic seizures in patients aged ≥12 years in Europe, the United States, and other countries, as well as a monotherapy treatment for focal onset seizures, with or without bilateral tonic–clonic evolution in patients aged 12 years and older in the United States.^2^, ^3^, ^4^, ^5^ It is well known that data from randomized controlled trials (RCT) must be evaluated cautiously, as they do not adequately reflect clinical practice. RCT data are rarely generalizable to populations with slightly different characteristics (e.g., patients in more severe or less severe clinical conditions, and special patients groups). Furthermore, simplified study designs do not allow thorough interpretation of data on interactions with concomitant treatments and on comorbidities.

Perampanel was reported as effective for the add‐on treatment of both generalized and focal seizures in several multicenter clinical trials.^2^, ^3^, ^4^, ^5^, ^6^ An excellent seizure control was described for focal seizures, especially for focal to bilateral tonic–clonic seizures.^7^ Some data suggest that PER is more effective when used as first add‐on therapy in patients with focal seizures rather than as second or later add‐on treatment, as shown by higher retention rates and efficacy data at 12 months.^8^, ^9^, ^10^, ^11^ This appears to be true also in selected sample, for example, temporal lobe epilepsy.^12^ The most common adverse events associated with PER include somnolence, fatigue, dizziness, headache, and irritability. They appear to be equally frequent in patients taking PER as a first add‐on or as a late add‐on.^10^, ^11^

We conducted a retrospective, observational study to evaluate the dose‐related therapeutic response of PER, as a reliable proxy for overall tolerability and effectiveness, in people with epilepsy (PWE) aged >12 years, having received PER as part of routine clinical care and as adjunctive treatment to a background monotherapy.

## 2 METHODS

This observational, multicenter, retrospective, longitudinal study involved patients with focal or generalized epilepsies recruited from 52 Italian epilepsy or neurology centers. The study was coordinated by the Regional Epilepsy Centre, Great Metropolitan Hospital “Bianchi‐Melacrino‐Morelli,” Reggio Calabria (see Appendix 1 for the complete list of participating Centres). Sites were preselected to ensure compatibility of their usual clinical records and visit schedule with study endpoints. Visit schedule included at least a baseline visit and three follow‐up visits approximately after 3 months (visit 1), 6 months (visit 2) and 1 year (visit 3). Inclusion Criteria were as follows: (a) diagnosis of epilepsy; (b) history of focal seizures, focal‐to‐bilateral seizures, or generalized tonic–clonic seizures (GTCS); (c) at least one seizure within the year prior to starting add‐on treatment; (d) treatment with PER as the only ASM added to a single concomitant ASM according to the usual clinical practice; (e) signature of a written informed consent by the patient or the legally authorized representative for the use of historical medical records (as required by the Independent Ethics Committee); (f) age > 12 years at study inclusion. Exclusion criteria were as follows: (a) PWE treated with 2 or more ASMs at the baseline visit; (b) patients enrolled in clinical trials of ASMs or medical devices during the period of retrospective observation; (c) inaccurate or unreliable clinical records according to the participating physicians; (d) patients with less than 3‐month follow‐up at the closing of the database. The study protocol was approved by the local Ethics Committee.

### 2.1 Procedure

Cases were identified by participating sites from the medical records of patients attending their epilepsy clinics. Data were obtained by reviewing medical records. The following data were collected for each patient at baseline: (a) date of assessment, date of patient informed consent for the use of personal historical medical records; (b) demographic data; (c) medical history: duration of epilepsy, classification of epilepsy (including syndrome and etiology, whenever possible), historical ASM treatment during the past 5 years, reason for discontinuation of previous ASM, reason for initiating PER, psychiatric history; (d) concomitant ASM: name of current ASM, and daily dose. Three, 6 months and 1‐year assessments included: (a) date of assessment; (b) patient's height and weight; (c) current PER dose, titration schedule used and dose of concomitant ASM; (d) seizure freedom since last evaluation; (e) adverse effects (open/general questions, not solicited for specific AEs). Final evaluation (if patient has dropped out of the follow‐up) included: (a) date of assessment; (b) patient's height and weight; (c) current PER dose, titration schedule used and dose of concomitant ASM; (d) seizure freedom (overall and by seizure type) since last evaluation; (e) AEs (open/general questions, not solicited for specific AEs). Seizure number at the different time intervals was collected retrospectively based on medical records. Tolerability was evaluated at every follow‐up by evaluating AEs directly referred by the patients and through specific questions about common AEs associated with PER.

### 2.2 Outcomes

The primary endpoint was the retention rate at 3, 6, and 12 months. To warrant data uniformity, all visits performed from 1.5 months to 4.5 months from baseline were considered as visit 1; all visits performed from 4.5 months to 9 months from baseline were considered as visit 2; all visits performed from 9 months to 15 months from baseline were considered as visit 3.

Secondary endpoints were efficacy and safety of PER therapy. Efficacy was assessed by quantifying changes in seizure frequency between the visits. We evaluated: the reduction in median number of seizures, normalized per 28 days; responders' rate (defined as a decrease in seizure frequency ≥ 50%); seizure freedom (no reported seizures since the previous observation); sustained seizure‐freedom and sustained seizure response, defined as a 100% and a ≥50% reduction in baseline seizure frequency that continued from the first time it was achieved through the 12‐month follow‐up without PER withdrawal. Retention time on PER was evaluated for all the PWE and by subgroups of patients according to the number of prior add‐on ASMs (0‐1, also defined as “early add‐on,” or >1), and by concomitant ASM (grouped by mechanisms of action).

Adverse effects were recorded verbatim, and coded using MedDRA.

### 2.3 Statistical analysis

Descriptive statistics were used: categorical variables were expressed as n. and percentages, and continuous variables were described as mean ± SD or median and interquartile range, as appropriate. Retention rates were calculated, at different time points, as the proportion of patients still receiving PER treatment. Data were analyzed by Chi‐square or *t*‐test as appropriate. Kaplan–Meier curves were built for time‐dependent analyses.

## 3 RESULTS

### 3.1 Whole sample

The study included 503 participants, aged 31.6 (IQR 19‐50.6) years. Demographic and clinical data are reported in Table 1. The median duration of epilepsy was 10 years, signifying that a large proportion of patients had a long‐standing history of epilepsy. About 80% had focal epilepsy. Structural and unknown etiology were represented almost equally. Among structural etiologies, malformations of cortical development and vascular etiology were the most frequent ones. PER was initiated for inefficacy of previous ASMs (75%) or for intolerable side effects of previous treatments (25%). About 20% of the patients presented a psychiatric comorbidity (mainly anxiety and depression). Of note, almost 200 (38.2%) PWE were previously treated with 0 or 1 add‐on ASMs (“early add‐on” group). Concomitant treatments included levetiracetam (27%), carbamazepine (16%), lamotrigine (12%), and valproate (11%) in most cases. The median target dose of PER was 4 mg/day, which was chosen for half of the patients. Only 15% of PWE were treated with 8 mg/day or more. The different titration schemes reflected the preferences of the treating physicians rather than tolerability or other patient‐specific issues, thus a specific analysis of effectiveness according to titration rate was not performed.

**TABLE 1:** Demographic and clinical data

	Whole cohort (n = 503)		Early add‐on (n = 192)	
	N	%	N	%
Sex (female/male)	295/208	59/41	109/83	57/43
Current age: median (IQR) years	31.6 (19.0‐50.6)	—	36.6 (19.7‐58.9)	—
Disease duration: median (IQR) years	10.1 (4.4‐19)	—	6.5 (2.3‐14)	—
History of febrile seizures	49	10	11	6
Previous epilepsy surgery	25	5	3	1.6
Psychiatric comorbidities	105	25	35	21
Type of epilepsy				
Focal	391	78	155	81
Generalized	96	19	36	18.5
Undetermined	16	3	1	0.5
Etiology of epilepsy: 9 pts missing				
Structural	207	41	89	47
Genetic	70	14	31	16
Unknown	213	42	70	37
Autoimmune	4	1	0	0
Number of previous add‐on ASMs (before concomitant): 2 pts missing				
0	52	10	52	27
1	140	28	140	73
2	115	23		
3	60	12		
4	52	11		
5	28	6		
6	15	3		
7	15	3		
8	11	2		
9	5	0.8		
10	5	0.8		
11	1	0.2		
14	1	0.2		
Target Perampanel daily dose (mg): 2 pts missing				
2	28	5.6	15	7.8
3	2	0.4	2	1.0
4	237	47	108	56.2
5	1	0.2	0	0
6	155	31	47	24.5
8	63	12.6	16	8.3
10	14	2.8	3	1.6
12	1	0.2	1	0.5
Titration scheme (intervals to next increase in dose): 1 pt missing				
1‐2 weeks	350	70	146	76
3‐4 weeks	137	27	44	23
5+ weeks	15	3	2	1
Concomitant ASM at baseline: 1 pt missing				
Brivaracetam	1	0.2	0	0
Carbamazepine	79	16	31	16
Clobazam	5	1	1	0.5
Clonazepam	4	0.8	1	0.5
Eslicarbazepine	8	1.6	1	0.5
Etosuxymide	1	0.2	0	0
Lacosamide	54	10.8	10	5.2
Levetiracetam	134	27	76	39
Lorazepam	1	0.2	1	0.5
Lamotrigine	62	12.4	21	11
Oxcarbazepine	48	10	18	9.4
Phenobarbital	12	2.4	7	3.7
Pregabalin	1	0.2	0	0
Phenytoin	4	0.8	0	0
Rufinamide	1	0.2	0	0
Topiramate	17	3.4	3	1.6
Vigabatrin	1	0.2	0	0
Valproic acid	56	11	20	10.5
Zonisamide	13	2.6	1	0.5
Concomitant ASM by mechanism of action: 1 pt missing				
Sodium blocker	192	38	60	31
GABA agonist	11	2	6	3
SV2A ligand	135	27	76	40
Various	164	33	49	26

Abbreviations: ASM, antiseizure medication; GABA, gamma amino butyrric acid; IQR, interquartile range; SV2A, synaptic vesicle 2A.

Retention rates for the whole cohort were 91%, 89%, and 89% at visit 1, visit 2, and visit 3. Visit 1 was performed by the whole study sample (503 subjects, according to inclusion criteria), visit 2 was performed by 382 (75.9%) subjects, and visit 3 by 249 (49.5%) subjects.

At baseline, median seizure number normalized per 28 days was 1.84 (IQR 0.92‐4.59; range 1‐300). Normalized median seizure number decreased to 0.77 (IQR 0‐2.5) at visit 1 (−64%), to 0.10 (IQR 0‐0.61) at visit 2 (−99%) and lastly to 0.07 (IQR 0‐0.5) at visit 4 (−99%). All differences were statistically significant from baseline. The difference between visit 2 and visit 1 was also significant, while the difference between visit 3 and visit 2 was not. This was due to very high efficacy data from the first months of PER treatment.

Responders' rate was also persistently high compared with baseline (57%, 82% and 84% at visits 1, 2 and 3). A significant proportion of PWE remained seizure free from all seizure types (31%, 50% and 49%, at 3, 6 and 12 months). Sustained 50% response was maintained for ≥6 and 12 months by 75% and 63% of patients. Sustained seizure freedom was maintained by 43% and 40% at 6 and 12 months.

**FIGURE 1:**
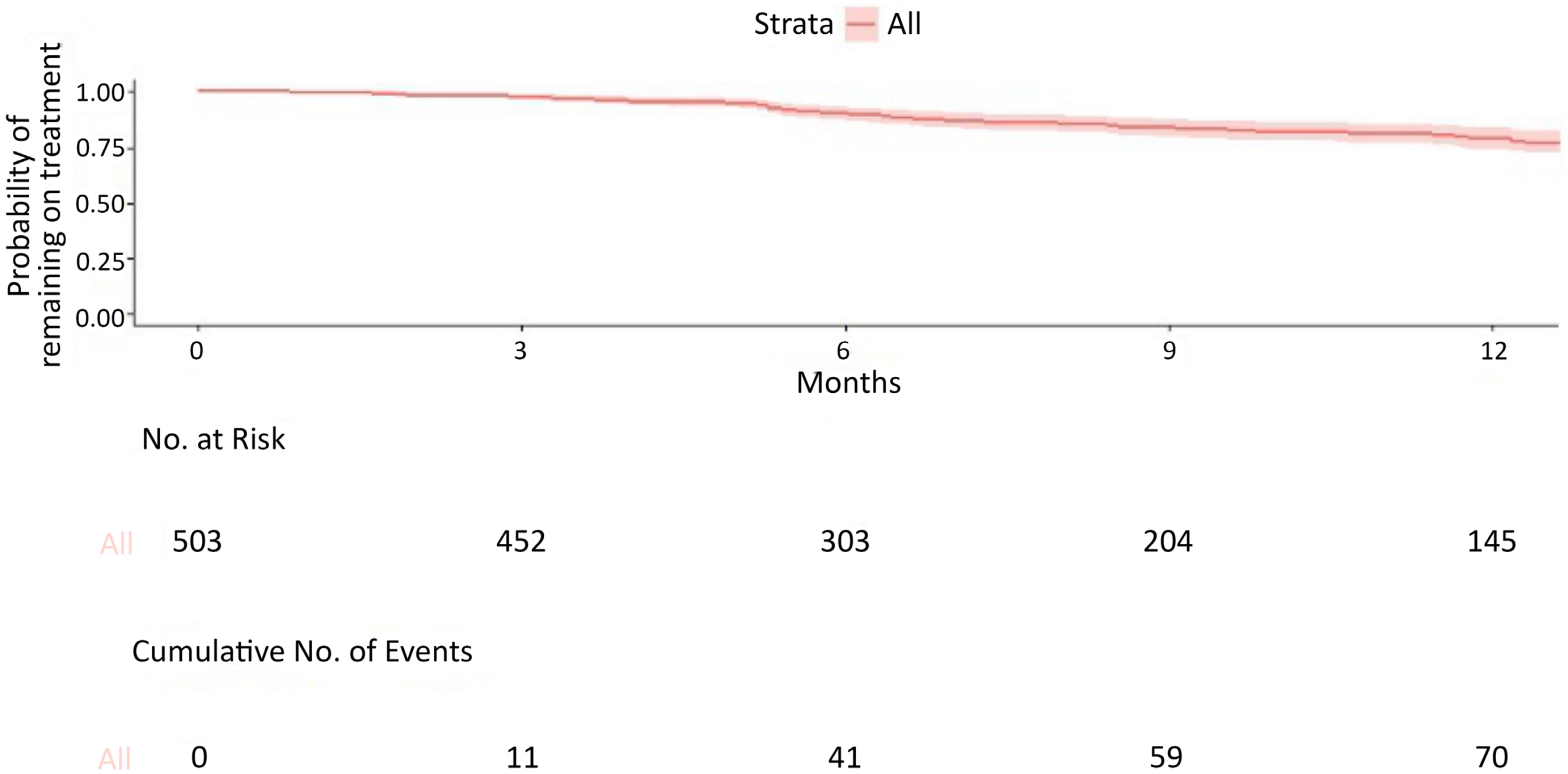
Kaplan–Meier curve depicting cumulative survival of the whole sample

Figure 1 shows the Kaplan–Meier curve of the overall retention time. The timeline was cut at 12 months. The cumulative probability to remain on treatment was 0.79 at 12 months.

As regards safety, data were available for 481 patients at visit 1, 288 patients at visit 2, and 114 patients at visit 3. Adverse events were reported by 25%, 21%, and 16% of patients, at visits 1, 2, and 3, respectively. Most were deemed to be related to PER use. Serious AEs were quite rare with a total of 15 events, with no reported deaths. In particular, nine patients withdrew for behavioral problems (irritability/aggressiveness), two for suicidal ideation, two for vertigo, and one each for myoclonus and fatigue. A small (7%) proportion of adverse events led to PER discontinuation. Allergic reactions were extremely rare (only three participants) and all occurred within the first 3 months of treatment. Dizziness/vertigo and behavior changes were the most frequent AEs at all time points. The details about adverse events are reported in Table 2. The rate and type of adverse events were similar in patients who took PER in combination with LEV, a drug that has been associated to a high risk of psychiatric adverse events.

**TABLE 2:** Adverse events of the whole population

Type of adverse event: n (%)	Visit 1 (3 months), n = 481	Visit 2 (6 months), n = 288	Visit 3 (12 months), n = 114
Drowsiness	16 (14%)	9 (15%)	3 (17%)
Dizziness/Vertigo	37 (31%)	12 (20%)	4 (22%)
Irritability/Nervousness/Insomnia	43 (36%)	24 (39%)	7 (39%)
Allergic reaction	3 (2.5%)	0	0
Other	19 (16.1%)	16 (26%)	4 (22%)
Total	118 (25%)	61 (21%)	18 (16%)
Serious adverse events	6 (5.1%)	9 (14%)	0
Withdrawal due to adverse events	30 (7%)	18 (6%)	8 (7%)

### 3.2 Early add‐on subgroup

**FIGURE 2:**
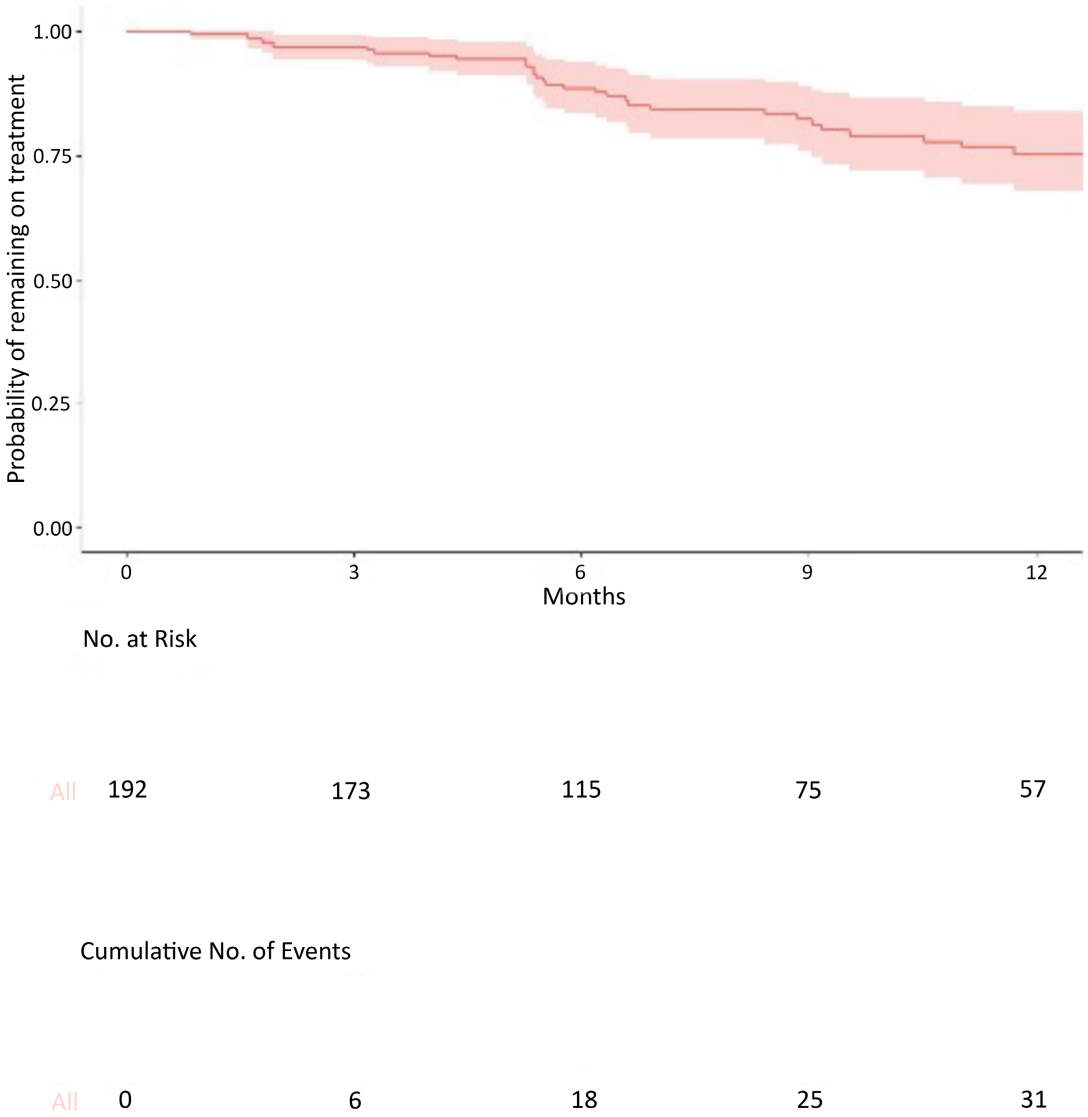
Kaplan–Meier curve depicting cumulative survival of the early add‐on group

A total of 192 subjects, (median age 36.6 years, IQR 19.7‐58.9), had received none or one add‐on ASM before the current therapy regimen. Demographic and clinical data are reported in Table 1. Almost 30% of these subjects received PER as their first add‐on ASM. The distribution of the different types of epilepsy and etiology did not differ between the early and the late add‐on groups (data not shown). Median target daily dose of PER was 4 mg. Retention rate was high in the early add‐on group (90%, 88%, and 90% at visits 1, 2, and 3, respectively). Data on visits 2 and 3 were available for 137 (71.5%) and 92 (47.9%) subjects, respectively. For efficacy analyses, we used only PWE and visits which were followed‐up according to study design, that is, at baseline and after 3, 6, and 12 months. Thus, data from 189 PWE were available at baseline, 102 (54%) at visit 1, 101 (53.4%) at visit 2, and 48 (25.4%) at visit 3. The normalized median seizure number was 1.84 per 28 days at baseline, 0.62 at visit 1 (−79% from baseline), 0.15 at visit 2 (−99% from baseline) and lastly 0.08 at visit 4 (−99% from baseline). All differences were statistically significant (*P* < .05) from baseline. The median seizure number reduction between visit 2 and visit 1 was also significant, while the difference between visit 3 and visit 2 was not. This was due to very high efficacy data from the first months of PER treatment. Responders' rate as compared with baseline was also persistently high (66%, 88%, and 83% at visits 1, 2, and 3, respectively). A significant proportion of PWE reached and maintained seizure freedom (42%, 50%, and 48%, at 3, 6 and 12 months, respectively) on PER add‐on treatment. Figure 2 shows the Kaplan–Meier curve of the retention time in the early add‐on group. The timeline was cut at 12 months. Of note, the cumulative probability to remain on treatment is very high in this population (over 0.75).

We also compared the early vs late add‐on groups in terms of PER daily dose, retention rate, and efficacy measures. Median PER daily dose was 4 mg in early add‐on group and 6 mg in late add‐on group, even though the most frequently chosen dose (modal value) was 4 mg/day even for late add‐on group. Retention rates were very similar in these two groups and the same was true for normalized median seizure number. Responders' rates were very similar at 6 and 12 months, while the early add‐on group showed a better responders' rate at 3 months (66% vs 53%, *P* = .05). Seizure freedom showed a similar trend, as the early add‐on group reached more easily seizure freedom at 3 months (42% vs 25%, *P* = .005). Adverse events rates were very similar to those reported for the whole group, with consistent discontinuation rates (data not shown).

### 3.3 Analysis by concomitant ASMs

The mechanisms of action of concomitant ASM were divided in three groups: sodium channel blockers (n = 192), SV2A ligands (n = 135), and others, including GABAergic, inhibitor of carbonic anhydrase, AMPA receptor blocker, and pleiotropic/unknown mechanisms (n = 175). GABAergic drugs were included in the “Other” mechanisms of action because of the limited number of patients taking that drug class. Detailed demographic and clinical data are reported in Table S1. No major differences among groups were observed in the primary or secondary endpoints, which were in line with the previously reported high effectiveness and efficacy. Early responders' rates and seizure freedom rates at 3 months were significantly lower in the sodium blocker group as compared to the other groups (*P* = .046 and .007).

## 4 DISCUSSION

We conducted a real‐life, retrospective, observational, multicenter study on PWE (both focal and generalized seizures) who were treated with PER as the only concomitant add‐on ASM, independently from the previous treatments. This study comprised 503 PWE, of whom a consistent portion of almost 200 subjects received PER as early (first or second) add‐on ASM. The data from the present study have to be compared with several retrospective studies on PER use in real‐life practice,^7^, ^8^, ^9^, ^10^, ^11^, ^12^, ^13^, ^14^, ^15^ with different characteristics.

The main strengths of this study are represented by the large sample size, by the use of PER in combination with a single ASM (thus limiting drug interactions) and by the representativeness of the population due to the real‐world setting. This allowed to explore the efficacy and safety of PER in a population whose features are similar to those encountered in clinical practice, including a proportion of patients with long‐standing epilepsy.

As witnessed by high retention rates, the treatment with PER was effective and well tolerated at 12 months. We showed a very high 12‐month retention rate of 90%, both in the whole sample and in the early add‐on group. This is in line with other studies that used add‐on PER to a monotherapy,^11^ but higher than most other reports.^10^, ^13^, ^14^, ^15^ This difference may be due to different study designs, including patients that had tried more ASMs before the current therapeutic regimen, or to different study populations (focal epilepsies only vs the inclusion of generalized epilepsies). Indeed, it is known that both efficacy and tolerability improve if PER is given as early add‐on and not as part of a complex drug regimen.^11^, ^13^, ^16^

Specific efficacy measures confirmed the usefulness of PER as single add‐on treatment. Median seizure number reduction and responders' rate were very high, in line with other studies, especially those with early use of PER.^8^, ^10^, ^11^ Indeed, seizure freedom rates were high both in the early and in the late add‐on groups. Of note, seizure freedom was reached within the first 3 months in the early add‐on group, while it took longer in patients previously treated with several ASMs. This was independent from baseline seizure frequency, that was identical in the two groups. Only another PER study^11^ analyzed seizure freedom rates at different time points in early vs late add‐on patients, finding similar seizure freedom rates at 3 months and higher efficacy in the early add‐on group at 12 months. These data are hard to compare, due to different inclusion criteria in the diverse study groups.^11^ Anyway, early PER efficacy with high seizure freedom rates at 3 months is undoubtedly relevant, as it may improve compliance and quality of life. Furthermore, the permanency of seizure freedom is a fundamental clinical parameter, and data from RCTs do not help in predicting long‐term response. In addition, intention‐to‐treat analysis, commonly used in regulatory trials, may overestimate seizure freedom rate due to short‐term exposure to investigational drugs.^17^ Sustained seizure freedom (and also sustained responders' rate) might represent better and more rigorous outcomes, also mitigating the effect of biases including placebo effect and regression of seizure frequency toward the median.

Throughout this observational study, PER median daily dose was 4 mg/day, lower than in most other studies.^10^, ^11^, ^14^, ^15^ This can depend on different populations (e.g., concomitant ASM or intrinsic responsiveness to treatment), on rigid study designs not allowing lower PER doses^8^, ^11^ or simply may reflect diverse prescribing attitudes in distinct countries. Indeed, in another Italian study^13^ the median PER dose was 4 mg/day in an early add‐on group, in line with our results.

In the present study, PER was effective in association with a variety of ASMs, without long‐term differences. We only observed a slightly lower efficacy (in terms of responders' rates and seizure freedom) at 3 months in PWE who received PER in association with sodium channel blockers. This may be due to the known pharmacokinetic interaction resulting in lower plasma levels of PER when the latter is administered together with CYP3A enzyme inducers as carbamazepine, oxcarbazepine, phenytoin, with the need to administer higher PER doses, which may require more time for titration.^18^

Adverse events were reported in about 20% of participants to the present study. This is in obvious contrast with RCTs reporting adverse events rates as high as 80%.^2^, ^3^, ^4^ This difference may be due to the recall bias in retrospective study design, the lower drug load of our patients, as well as the low doses and the slow, customized titration, which were likely to result in improved tolerability. Interestingly, almost all adverse events were deemed related to PER by treating physicians. Real‐world studies report a prevalence of adverse events ranging from 15% to 80%.^11^, ^12^, ^13^, ^14^, ^15^ These data are hardly comparable due to different inclusion criteria, PER doses and titration schemes. No new or unexpected safety concerns emerged over this long‐term study using PER as the only concomitant ASM.

The main limitations of this study are represented by the retrospective design and by the exclusion of PWE with incomplete data or with visits not respecting the time schedule established. These drawbacks may limit the generalizability of the results. Moreover, the large number of early add‐ons and the “simple therapeutic regimen,” which represent the strengths of the study, probably affect the high retention rates and seizure freedom. Another limitation may be represented by the relatively low number of patients reaching 12‐month follow up (about 50% of the initial sample), which limits the strength of the data on long‐term effectiveness. Of note, in other studies with similar design^11^, ^12^, ^14^ the rate of patients who did not achieve 12‐month follow‐up was comparable to our study, reflecting a limitation common to real‐world studies.

In conclusion, in our large sample real‐world study, PER therapy was maintained for 1 year by most patients. The effectiveness was excellent and was maintained over time until 1‐year follow‐up, with a high seizure freedom rate. In the “early add‐on” group, these study results were consistent, with an early efficacy observed even at 3 months. Tolerability was good, with a low rate of PER‐related adverse events.

## FUNDING INFORMATION

The study was funded by Eisai srl.

## CONFLICT OF INTEREST

Prof. Umberto Aguglia (speaker honoraria from Eisai); Dr Fedele Dono (travel support and speaker honoraria from EISAI); Dr. Alfredo D'Aniello (Research grant for UCB Pharma and Speaker/Honoraria for Eisai, UCB Pharma, Angelini Pharma and Lusofarmaco); Dr. Giancarlo Di Gennaro (speaker honoraria from EISAI, UCB‐Pharma, Livanova, Lusofarmaco, GW Pharmaceuticals. Served on advisory boards for Bial, Arielle therapeutics, Angelini Pharma); Prof. Edoardo Ferlazzo (speaker honoraria from UCB, Eisai, Angelini); Dr. Pasquale Striano (P.S. speaker honoraria and participated on advisory boards for Biomarin, Zogenyx, GW Pharmaceuticals, research funding by ENECTA BV, GW Pharmaceuticals, Kolfarma Srl). All other authors have no conflicts of interest to declare. We confirm that we have read the Journal's position on issues involved in ethical publication and affirm that this report is consistent with those guidelines.

## Supporting information


Table S1

## APPENDIX 1

### MEMBERS OF THE PEROC STUDY GROUP (IN ALPHABETICAL ORDER)

Arbasino Carla (Voghera, Pavia, Italy), Bagnasco Irene (Torino, Italy), Barbero Pierangelo (Torino, Italy), Bartolini Emanuele (Prato, Italy), Bassetti Maria A. (Roma, Italy), Belcastro Vincenzo (Lodi, Italy), Beretta Simone (Monza, Italy), Boero Vanni (Taranto, Italy), Bonuccelli Alice (Pisa, Italy), Caggia Emanuele (Ragusa, Italy), Cantello Roberto (Novara, Italy), Casellato Susanna (Sassari, Italy), Casula Natascia (Mestre, Venezia, Italy), Cesnik Edward (Ferrara, Italy), Ciampa Clotilde (Napoli, Italy), Cipriani Anna Maria (Roma, Italy), Coletti Moja Mario (Torino, Italy), Conti Marta (Sassari, Italy), Coppola Giangennaro (Salerno, Italy), Corea Francesco (Foligno, Perugia, Italy), Crichiutti Giovanni (Udine, Italy), d'Orsi Giuseppe (Foggia, Italy), Dainese Filippo (Venezia, Italy), Danieli Alberto (Conegliano Veneto, Treviso, Italy), De Curtis Marco (Milano, Italy), De Giorgis Valentina (Pavia, Italy), Del Gaudio Luigi (Catellammare di Stabia, Napoli, Italy), Deleo Francesco (Milano, Italy), Di Gennaro Giancarlo (Pozzilli, Isernia, Italy), Giacomo Evangelista (Chieti, Italy), Filipponi Stefania (Trento, Italy), Fortunato Francesco (Catanzaro, Italy), Gagliano Attilio (Cuneo, Italy), Giacomini Thea (Genova, Italy), Gilio Francesca (Roma, Italy), Giordano Alfonso (Napoli, Italy), Giuliano Loretta (Catania, Italy), Haggiag Shalom (Roma, Italy), Jensen Stella (Massa, Massa‐Carrara, Italy), Labate Angelo (Messina, Italy), Lupato Angelica (Legnago, Verona, Italy), Macorig Greta (Gorizia, Italy), Mancardi Maria Margherita (Genova, Italy), Marino Daniela (Arezzo, Italy), Marrelli Alfonso (L'Aquila, Italy), Mostacci Barbara (Bologna, Italy), Muccioli Lorenzo (Bologna, Italy), Negri Susanna (Conegliano Veneto, Treviso, Italy), Operto Francesca (Salerno), Orsini Alessandro (Pisa, Italy), Palumbo Pasquale (Prato, Italy), Pascarella Mariagrazia (Lodi, Italy), Peretti Alessia (Vicenza, Italy), Perri Gabriella (Garbagnate, Milano, Italy), Pondrelli Federica (Bologna, Italy), Pustorino Giuseppina (Foggia, Italy), Ranzato Federica (Vicenza, Italy), Servo Serena (Cuneo, Italy), Siri Laura (Genova, Italy), Spitaleri Orazio (Acireale, Catania, Italy), Stabile Andrea (Monza, Italy), Strigaro Gionata (Novara, Italy), Tinuper Paolo (Bologna, Italy), Varesio Costanza (Pavia, Italy).
