# Supplementary material for: Effectiveness of perampanel as the only add‐on: Retrospective, multicenter, observational real‐life study on epilepsy patients
Source: Epilepsia Open. 2022 Sep 22;7(4):687–96. doi: 10.1002/epi4.12649 (PMC9712478; doi:10.1002/epi4.12649)
Supplement: Supplementary file 1 — Table S1 [file EPI4-7-687-s001.docx]

|  | **Sodium blocker (n=192)** | **SV2A** ligand **(n=135)** | **Other (n=175)** |
| --- | --- | --- | --- |
| Female sex: n (%) | 101 (53%) | 74 (55%) | 117 (68%) |
| Current age: median (IQR) years | 36 (21-50) | 31 (19-56) | 26 (17-46) |
| Disease duration: median (IQR) years | 12 (6-22) | 5.07 (1.8-13) | 11 (6-18) |
| History of febrile seizures | 18 (9%) | 7 (5.2%) | 28 (14.2%) |
| Previous epilepsy surgery | 13 (7%) | 5 (3.7%) | 11 (4%) |
| Psychiatric comorbidities | 41 (25%) | 19 (16.1%) | 48 (32.6%) |
| Type of epilepsy | | | |
| - Focal | 184 (96%) | 96 (71%) | 107 (61%) |
| - Generalized | 5 (2.4%) | 35 (26%) | 57 (33%) |
| - Undetermined | 3 (1.6%) | 4 (3%) | 9 (6%) |
| Etiology of epilepsy | | | |
| - Structural | 89 (46.5%) | 63 (47%) | 54 (30%) |
| - Genetic | 12 (6%) | 22 (16%) | 38 (21%) |
| - Unknown | 91 (47.5%) | 50 (37%) | 83 (49%) |
| Target Perampanel daily dose (mg) | | | |
| 2 | 5 (2.6%) | 9 (6.7%) | 18 (8%) |
| 3 | 0 | 1 (0.7%) | 0 |
| 4 | 76 (39.3%) | 75 (56%) | 84 (49%) |
| 6 | 63 (33%) | 38 (27.6%) | 54 (32%) |
| 8 | 36 (18.8%) | 10 (7.5%) | 17 (10%) |
| 10 | 11 (5.8%) | 2 (1.5%9 | 2 (1%) |
| 12 | 1 (0.5%) | 0 | 0 |
| Titration scheme (intervals to next increase in dose) | | | |
| 1-2 weeks | 132 (69%) | 105 (78%) | 112 (64%) |
| 3-4 weeks | 54 (28%) | 25 (18.5%) | 58 (33%) |
| 5+ weeks | 6 (%) | 5 (3.5%) | 5 (3%) |

**Supplementary Table.** Demographic and clinical data of subgroups in function of mechanism of action of concomitant ASM. IQR: interquartile range.
